# Giant Up-Conversion Efficiency of InGaAs Quantum Dots in a Planar Microcavity

**DOI:** 10.1038/srep03953

**Published:** 2014-02-04

**Authors:** Qinfeng Xu, Carlo Piermarocchi, Yuriy V. Pershin, G. J. Salamo, Min Xiao, Xiaoyong Wang, Chih-Kang Shih

**Affiliations:** 1 National Laboratory of Solid State Microstructures and School of Physics, Nanjing University, Nanjing 210093, China; 2 Department of Physics, the University of Texas at Austin, Austin, TX 78712, USA; 3 Department of Physics and Astronomy, Michigan State University, East Lansing, MI 48824, USA; 4 Department of Physics and Astronomy and USC Nanocenter, University of South Carolina, Columbia, SC 29208, USA; 5 Department of Physics, University of Arkansas, Fayetteville, AR 72701, USA; 6 Department of Physics and Optoelectronic Engineering, Ludong University, Yantai 264025, China

## Abstract

Self-assembled InGaAs quantum dots (QDs) were fabricated inside a planar microcavity with two vertical cavity modes. This allowed us to excite the QDs coupled to one of the vertical cavity modes through two propagating cavity modes to study their down- and up-converted photoluminescence (PL). The up-converted PL increased continuously with the increasing temperature, reaching an intensity level comparable to that of the down-converted PL at ~120 K. This giant efficiency in the up-converted PL of InGaAs QDs was enhanced by about 2 orders of magnitude with respect to a similar structure without cavity. We tentatively explain the enhanced up-converted signal as a direct consequence of the modified spontaneous emission properties of the QDs in the microcavity, combined with the phonon absorption and emission effects.

Recent advancement of nanostructure fabrication has opened many new scientific and technological frontiers, one of which is the subject of cavity-QED (cavity quantum electrodynamics) with solid-state artificial atoms such as semiconductor or superconductor quantum dots (QDs). Indeed, modification of the spontaneous photon emission (the Purcell effect)^1^,^2^ and multiple photon emission and re-absorption (vacuum Rabi oscillations)^3^,^4^,^5^ have been achieved in QD systems. All these cavity-QED works focused on the interaction between the cavity photonic modes and the electronic states of QDs, similar to that of natural atoms. It is noted that there are other known solid state systems where the cavity-QED effect interacts with lattice phonons. For example, quantum well excitons in a planar microcavity are subject to the cavity-bottleneck effect, resulting in an accumulation of the optical excitations in a particular region of the energy-momentum dispersion^6^. This accumulation, or bottleneck, plays a key role in polariton Bose condensation^7^, parametric amplification^8^, and lasing^9^ behaviors.

Up-converted (UC) photoluminescence (PL) in semiconductors describes an intriguing optical process whereby the emission energy is higher than that of the excitation photons. The extra energy needed for UC PL can be acquired through a variety of mechanisms, such as thermal activation assisted by phonons^10^,^11^, Auger processes involving multiple electron-hole pairs^12^,^13^, and two-photon absorption via either virtual^14^ or intermediate^13^,^15^ states. However, a common feature is that the UC process is always associated with a significantly lower efficiency as compared to its down-converted (DC) counterpart. Here, we investigate how lattice vibrations, the quantum degree of freedom that distinguishes semiconductor QDs from natural atoms, manifest in a QD - planar cavity system. In particular, the interplay of lattice phonons, cavity photons and QD excitons can lead to a giant UC efficiency of InGaAs QDs (2 orders of magnitude enhancement with respect to a similar structure without cavity). This result represents an important step towards the realization of new solid state devices in which cavity-QED could be used to control carrier-lattice thermalization processes.

## Results

### Sample structure and PL characterizations

**Figure 1:**
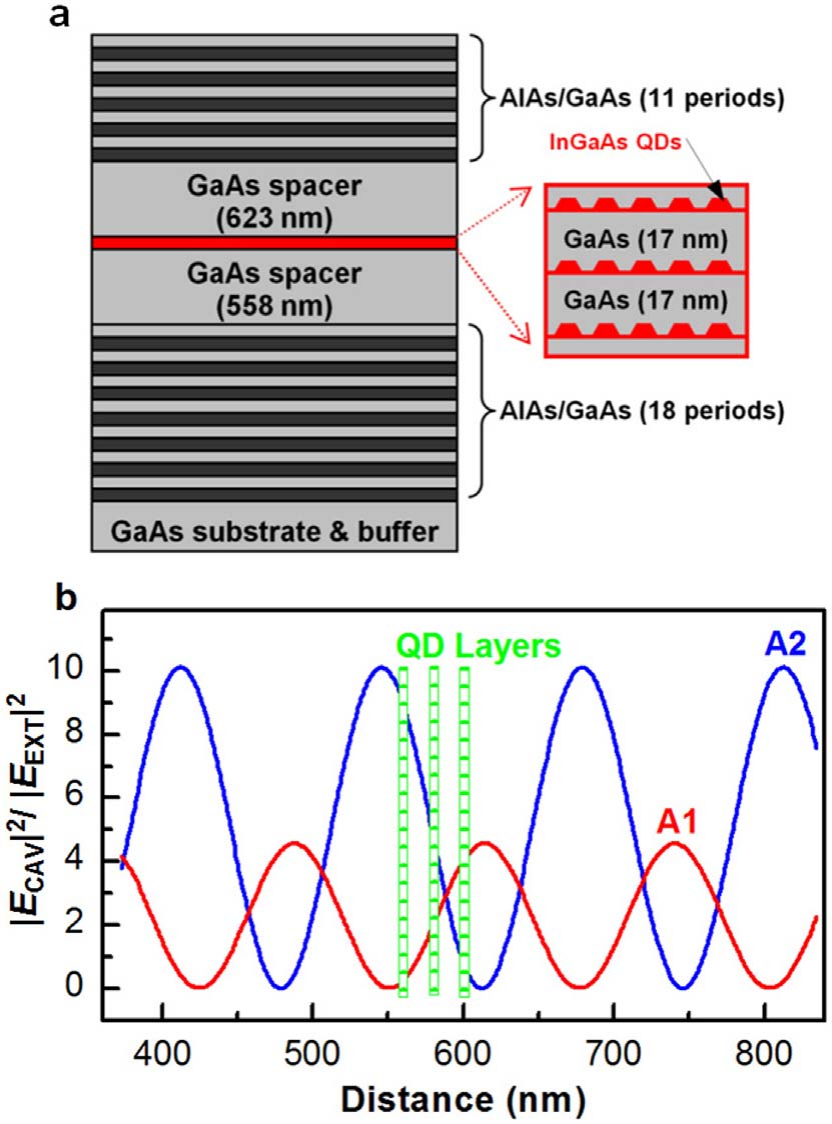
Sample structure and electric field profile. (a), The microcavity structure with three layers of InGaAs QDs embedded between the bottom and top mirrors. (b), Theoretically calculated intensity profiles for the electric fields of vertical cavity modes A1 and A2 in the cavity regions where the three layers of QDs are located. The horizontal axis stands for the distance measured from the bottom cavity mirror. *E*_CAV_ (*E*_EXT_): electric field inside (before getting into) the cavity.

**Figure 2:**
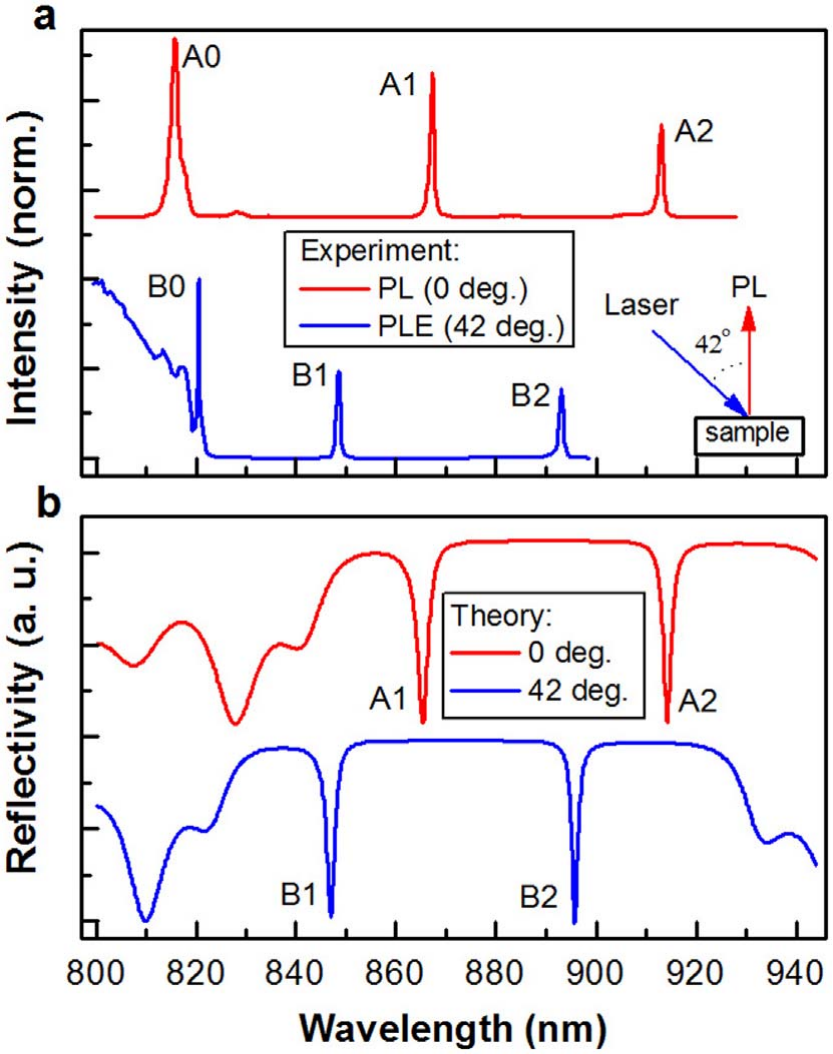
Vertical and propagating cavity modes. (a), PL spectrum excited with laser at 780 nm and PLE spectrum monitored at the cavity mode position of A2 with the laser incident angle of ~42°. Both the PL and PLE spectra were measured at ~8 K and normalized to their respective maximum intensities. Inset: Schematic of the experimental setup. (b), Theoretically calculated reflectivity curves for photons propagating along the 0° and 42° directions.

The structural configuration of the InGaAs QD - planar cavity sample is schematically shown in Fig. 1a and the detailed fabrication procedures were previously reported elsewhere^16^ (see Methods). Using the laser excitation and fluorescence collection scheme in the bottom-right inset of Fig. 2a, we measured time-integrated PL spectrum of the sample at ~8 K with an excitation laser wavelength of 780 nm. As shown in Fig. 2a, the two PL peaks labeled A1 (at ~865.3 nm) and A2 (at ~914.2 nm) are due to QD emissions coupled to two vertical cavity modes. These A1 and A2 peak positions are in excellent agreement with those obtained from a theoretical transfer-matrix calculation^17^ of the reflectivity that includes the details of the cavity structures (Fig. 2b). In Fig. 1b, we also show the calculated intensity profiles of the electric fields for the A1 and A2 modes at the cavity regions. The coupling between the three layers of QDs and the cavity modes is larger for A2, which will be taken into account in the analysis of the Stokes and anti-Stokes intensities described later in the text.

The planar cavity geometry also strongly influences the PL excitation (PLE) spectrum of the InGaAs QDs. As shown in Fig. 2a, the PLE spectrum measured for A2 emission with the laser incident angle at *θ* = 42° have resonances at B1 and B2 with shorter wavelengths than those of A1 and A2, respectively. These B1 and B2 peaks are attributed to the propagating modes (at an angle *θ* with respect to the normal direction) whose wavelength *λ_a_* can be expressed as 
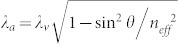
, where *n_eff_* is the effective refractive index of the microcavity sample, and *λ_v_* is the wavelength of the vertical cavity mode. The reflectivity curve theoretically calculated for light propagating in the *θ* = 42° direction is also shown in Fig. 2b, and the two vertical cavity modes A1 and A2 are now replaced by their blue-shifted angular counterparts of propagating modes B1 and B2, respectively. Indeed, the calculated propagating modes show excellent agreements with the observed B1 (at ~846.9 nm) and B2 (at ~895.7 nm) peaks in the PLE spectrum. Based on the two reflectivity curves calculated in Fig. 2b, the peak A0 (B0) observed at ~815.8 nm (~820.5 nm) in the PL (PLE) spectrum of Fig. 2a can be assigned to the GaAs emission (absorption) modulated by the edge of the cavity stop band.

### Cavity-enhanced UC PL

**Figure 3:**
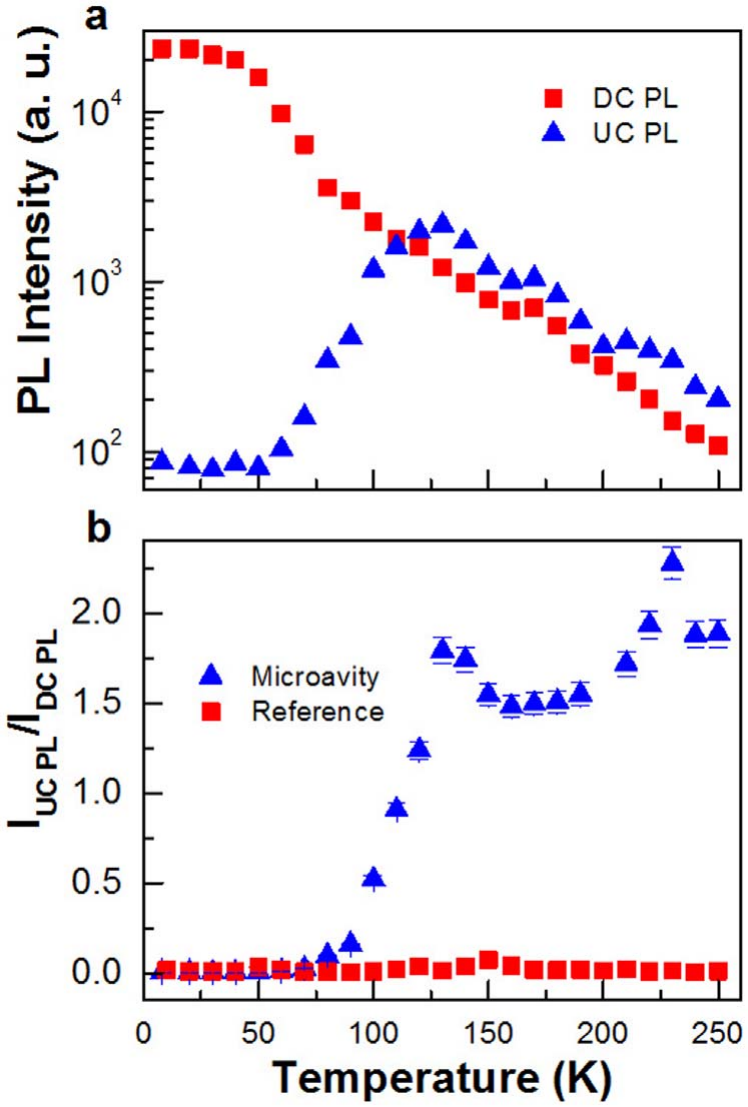
Temperature-dependent PL measurements. (a), Temperature dependences of the DC PL (excited at B1) and UC PL (excited at B2) intensities of A1 for the microcavity sample. (b), Temperature dependences of the UC to DC PL intensity ratios for both the microcavity and reference samples.

**Figure 4:**
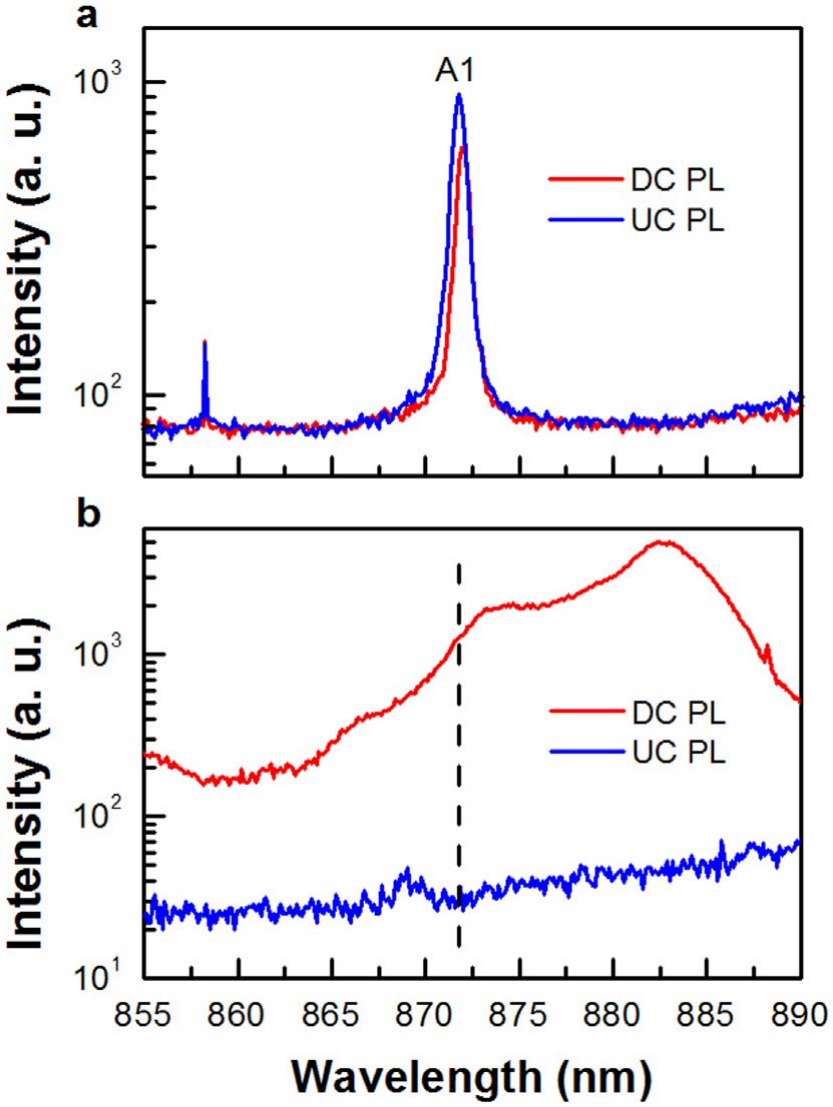
Comparisons of the DC and UC PL spectra. (a), DC and UC PL spectra of the microcavity sample excited at ~120 K with the laser wavelengths set at the propagating cavity mode positions of B1 and B2, respectively. (b), Same PL measurements with the reference sample, where the dashed vertical lines in the middle of the two PL spectra marked the vertical cavity mode position of A1 in the microcavity sample.

Because of the special cavity design that accommodates two cavity modes both coupled to the QD layers, we can excite QD states coupled to A1 either with laser at B1 to get the DC PL, due to a Stokes process, or with laser at B2 to study the UC PL, due to an anti-Stokes process. As can be seen from Fig. 3a, the DC PL intensity of A1 excited at B1 keeps decreasing with the increasing temperature, which can be explained by the involvement of nonradiative decay due to thermal excitation of carriers from InGaAs QDs to the surrounding GaAs barrier. For comparison, the UC signal excited at B2 becomes sizeable only at ~40 K and its PL intensity gradually increases with the increasing temperature, reaching a maximum at ~120 K. Most intriguingly, as can be seen from Fig. 4a, the UC PL intensity of A1 at this temperature is even larger than that of its DC PL when excited with the same laser power (see Fig. S-1 for the same PL spectra plotted at a larger wavelength range). This temperature-dependent behavior of the UC PL below ~120 K strongly implies that another mechanism, which we attribute to the cavity-enhanced effect later in the text, is dominant over the nonradiative decay at this temperature range. When the temperature is further increased from ~120 K, the nonradiative decay takes over, leading to the continuously deceasing intensity of the UC PL. However, as shown in Fig. 3b, the UC to DC PL intensity ratio *I_UC_*/*I_DC_* always exceeds unity for the temperatures above ~120 K (see Fig. S-2 for the same figure plotted on a semi-logarithmic scale).

The absence of UC PL at low temperature and the linear dependence of its intensity on the laser power density (Fig. S-3) can easily exclude such mechanisms as the two-step two-photon absorption and Auger processes^13^,^15^ previously reported in the UC experiments with self-assembled QDs. In order to investigate the physical origin of the strong UC PL signal at A1, we have carried out the same temperature-dependent measurements on a reference sample with the same bottom mirror and QD structures but without the top mirror (see Fig. S-4 for the PL spectrum of the reference sample). As shown by the vertical dashed line in Fig. 4b, the UC PL intensity (excited at B2) measured for A1 at ~120 K is about two orders of magnitude weaker than that of its DC PL (excited at B1). In Fig. 3b, we plot the temperature dependence of *I_UC_*/*I_DC_* for the reference sample, which is always significantly lower than that of the microcavity sample measured at any given temperature. In fact, this low intensity ratio between the UC and DC PL in the reference sample is commonly encountered in many UC PL studies of semiconductor nanocrystal QDs and can be well explained by a thermal excitation model^18^,^19^. Thus, the anomalously enhanced *I_UC_*/*I_DC_* observed here in the microcavity sample should be closely related to the existence of the cavity structure.

As shown in Fig. 3a and discussed earlier in the text, the nonradiative decay is dominant over other mechanisms after ~120 K, and both the UC and DC PL signals approach the background level after ~200 K, making it difficult to get a reliable UC to DC PL intensity ratio. Based on the above two reasons, in Fig. 3b we plot the temperature dependences of *I_UC_*/*I_DC_* of A1 for the microcavity and reference samples only up to ~250 K in order to establish a simple model without considering the nonradiative decay effect. In the reference sample, the intensity ratio is consistent with the thermal excitation model for temperatures around ~100 K. In the microcavity case, the UC PL is dramatically enhanced, being even stronger than the DC PL above ~120 K. This enhanced UC PL could be attributed to different QD density of states at B1 and B2, which would add a constant factor to the *I_UC_*/*I_DC_* intensity ratio. However, the QD density of states is the same for the microcavity and reference samples both containing QDs with nominally the same parameters and grown under the same conditions. In the cavity case, the respective electric field profiles of B1 and B2, which are similar to those of A1 and A2, dictate that the coupling strengths between these two modes and the three QD layers should be different. From Fig. 1b, we can see that this correction, when averaged over the three QD layers, increases the UC signal only by about a factor of 2, which is much smaller than the observed enhancement of 2 orders of magnitude.

### Theoretical modeling

**Figure 5:**
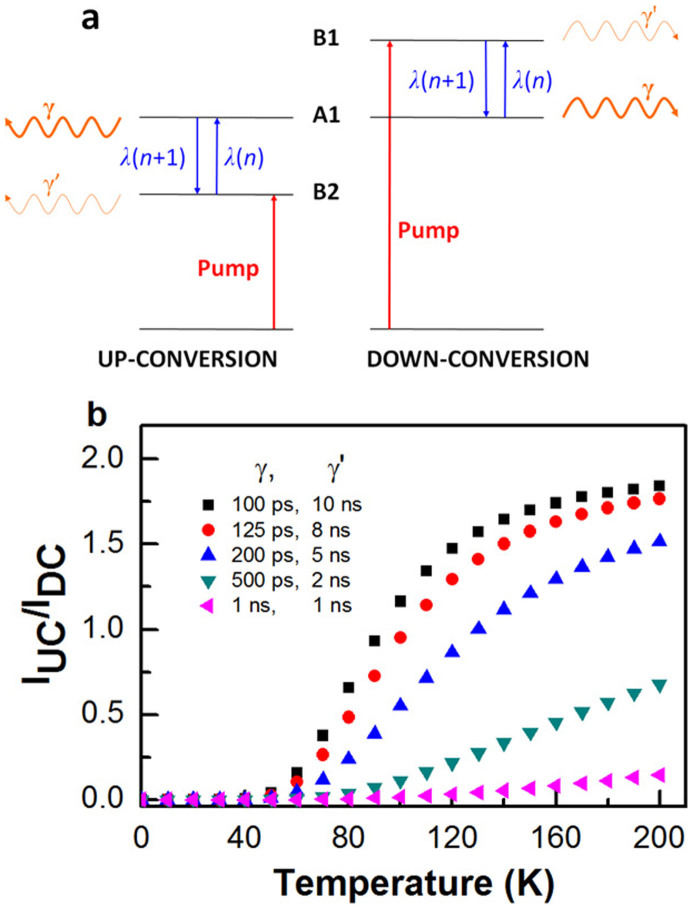
Theoretical model and calculations. (a), Schematic of the rate equation model describing the UC (left) and DC (right) PL processes. The levels A1 and B2 in the UC process and the levels B1 and A1 in the DC process are exciton states from different QDs. (b), Temperature-dependent UC to DC PL intensity ratios calculated with a phonon scattering rate *λ*^−1^ = 1 ps, the spontaneous emission rates *γ*^−1^ = *γ*′^−1^ = 1 ns for the reference sample, and a range of their enhancement/suppression factors at 2, 5, 8 and 10, corresponding to *γ*/*γ*′ = 4, 25, 64, and 100, respectively, in the cavity case.

We tentatively explain the enhanced UC signal as a direct consequence of the modified spontaneous emission properties of the QDs in the microcavity, combined with the phonon absorption and emission effects. We have looked for the LO (longitudinal optical) phonon resonance of InGaAs at ~33 meV by tuning the laser incident angle *θ* at ~120 K, and therefore changing the excitation laser wavelength and the energy separation between A1 and B2. Within the range of ~20–40 meV, we did not observe any considerable change in the UC PL intensity of A1 (Fig. S-5). The lack of the LO phonon resonance implies that a broad-band phonon spectrum might be formed at this temperature with both LO and acoustic phonons participating in the UC PL process^20^. In the UC process (left panel, Fig. 5a), the pump excites QD transitions at the energy B2, which corresponds to a large-angle cavity mode and is associated with a spontaneous emission rate *γ*′ for photo-excited excitons. Meanwhile, these excitons can be thermally activated into the A1 energy position by means of phonon absorption to yield the UC signal. Since excitons at A1 are now resonant with a vertical cavity mode, they would recombine with a larger spontaneous emission rate of *γ* > *γ*′. In the DC process (right panel, Fig. 5a), the pump excites QD transitions at the energy B1 and photo-excited excitons would either emit photons there or decay to the A1 energy position by phonon emission. Similar to the UC case, the large-angle mode B1 and vertical mode A1 have spontaneous emission rates of *γ*′ and *γ*, respectively. We treat the UC and DC processes independently and solve their respective rate equations analytically to get the time-integrated PL emissions of *I_UC_* and *I_DC_* at A1 (see Supplementary Information). The UC to DC PL intensity ratio *I_UC_*/*I_DC_* for the light emitted at A1 is then given by 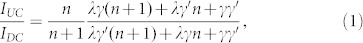
where *λ* is the exciton-phonon coupling constant, and 
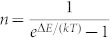
 is the phonon occupation number at the energy needed in the emission and absorption processes. Here, *k* is the Boltzmann constant, *T* is the temperature, and Δ*E* is the energy separation between the two energy levels involved in the UC or DC process. Equation (1) would be reduced to the thermal ratio 
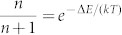
 in the limit of *γ* = *γ*′, while for *γ* > *γ*′ there is always a temperature-dependent enhancement of *I_UC_*/*I_DC_*, which can be intuitively understood as an increase of the pumping efficiency in the UC case. In the DC process, photo-excited excitons reach the short lifetime states at A1 by phonon emission, which is always efficient at low temperature, and recombine quickly by photon emission. On the other hand, in the UC process, excitons have to absorb phonons first before reaching the short lifetime states at A1. This process is weak at low temperature, and an exciton population tends to accumulate at B2. Overall, more excitons would be stored in the cavity structure in the UC than in the DC steady-state pumping configuration.

The calculated results of the above model are plotted in Fig. 5b, where we have used a phonon scattering rate *λ*^−1^ = 1 ps^20^, the spontaneous emission rates *γ*^−1^ = *γ*′^−1^ = 1 ns for the reference sample, and a range of their enhancement/suppression factors at 2, 5, 8 and 10, corresponding to *γ*/*γ*′ = 4, 25, 64, and 100, respectively, in the cavity case. The factor 2 due to different coupling strengths between the two cavity modes and the three QD layers was also included in the theoretical calculation. It can be clearly seen that, so long as there is cavity modification of the spontaneous emission rates, *I_UC_*/*I_DC_*would deviate obviously from the thermal ratio of 
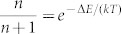
. Moreover, for all the cavity *γ*/*γ*′ values used in the calculations, a saturation effect of *I_UC_*/*I_DC_* is universally observed above the temperature of ~120 K. This agrees well with the turning point of *I_UC_*/*I_DC_* observed in Fig. 3b at almost the same temperature, beyond which its decreasing trend should arise from the nonradiative decay of thermally activated excitons. Overall, the giant UC efficiency shown in Fig. 3b can be well reproduced from our theoretical calculations with *γ*/*γ*′ > 25 in Fig. 5b.

## Discussion

It is common to have two/three orders of magnitude enhancement in the angular dependence of the spontaneous emission rate of quantum well excitons in 2D microcavities^21^,^22^. Moreover, it was previously demonstrated in an InGaAs QD - planar cavity system similar to ours that the spontaneous emission rates of QDs are strongly dependent on the detuning between their emission energy and the vertical cavity mode^23^. Specifically, it was shown in ref. 23 that the emission rates from QDs resonant with the wave vector *k* = 0 cavity mode are about 5 times larger than those from QDs resonant with large angle modes, which would imply *γ*/*γ*′ ~ 5 (see Fig. S-6 for the PL decay curves of our samples). While this value of *γ*/*γ*′ is too small according to our model to explain the observed behavior in our sample, the discrepancy can be attributed to many factors. First of all, while similar, our structure contains different mirrors and a different number of QD layers, which have a different size distribution and density. In addition, we have interpreted our experimental results using a simplified theoretical model. In particular, the model is not considering the spatial distribution of the excitons on different QDs, so it cannot distinguish between intra- and inter-QD transitions. The effect of a planar cavity on the radiative and phonon-assisted energy transfer among two QDs has recently been studied theoretically in ref. 24. A full microscopic theoretical description taking into account the radiative and phonon-assisted energy transfer in an ensemble of multi-level QDs has not yet been addressed theoretically and is beyond the scope of this paper. However, the simple model in Fig. 5 indicates clearly that cavity enhanced/suppressed emission can lead to strong deviations from the thermal ratio of UC *versus* DC PL that are in agreement with our experimental observations.

To summarize, we have shown that a QD system attains a giant UC efficiency when placed in a planar microcavity. This phenomenon was explained by the interplay of cavity-induced enhancement/suppression of the emission rate combined with the phonon absorption and emission. This result could potentially guide the design of new solid state geometries for novel cavity-QED effects to realize more efficient energy flow in nanostructures. Such control of energy flow could be important to optimize devices aimed at collecting and emitting light such as in QD-based solar cells and light-emitting diodes.

## Methods

### Sample preparation

The microcavity structure was fabricated by molecular beam epitaxy on a (100)-oriented GaAs substrate^16^. Three layers of InGaAs/GaAs QDs are confined between distributed Bragg reflectors (DBRs) consisting of 11 (top) and 18 (bottom) pairs of GaAs/AlAs quarter-wave layers. For comparison, a reference sample was also fabricated with the same bottom DBR and QD structures but without the top DBRs. The QD density in each layer is ~5 × 10^10^ cm^−2^ and the average QD size is ~40 nm.

### Optical measurements

The samples were mounted in a helium flow cryostat with variable temperatures from ~8–300 K. PL and PLE measurements were performed using a 1 ps pulsed Ti-sapphire laser with an 82 MHz repetition rate and a wavelength tuning range of ~720–950 nm. The laser beam was focused onto the sample surface with a typical power density of ~50 W/cm^2^ and at an incident angle of ~42° unless otherwise specified in the text. PL from the samples was collected vertically by a microscope objective and sent through a 0.5 m spectrometer to a charge-coupled device camera or an avalanche photo diode for the time-integrated and time–resolved optical measurements, respectively.

Mainly due to the refractive index change with the increasing temperature, peak wavelengths of the vertical cavity modes A1 and A2 increase slowly at the speeds of ~0.068 nm/K and ~0.056 nm/K, respectively. This also leads to the red shifts in the peak wavelengths of their respective angular counterparts B1 and B2. To obtain the DC (UC) PL from the ensemble of QDs coupled to A1, at each temperature, the excitation laser wavelength was adjusted to the new B1 (B2) position as judged by the maximum DC PL intensity from A1 (A2). For the fixed laser incident angle *θ* = 42°, the energy difference between B1 and A1 (A1 and B2) falls within the range of ~25–40 meV for all the temperatures scanned in the DC and UC PL measurements.

## Author Contributions

X.W. and C.K.S. conceived and designed the experiments. G.S. provided the samples. X.W. and Q.X. performed the optical experiments. C.P., Y.P. and Q.X. did the theoretical calculations. X.W., C.P., M.X. and C.K.S. co-wrote the manuscript. All authors have contributed to the interpretations of the data and commented on the manuscript.

## Supplementary Material

Supplementary Information Supplementary Information
