# Supplementary material for: Giant Up-Conversion Efficiency of InGaAs Quantum Dots in a Planar Microcavity
Source: Sci Rep. 2014 Feb 4;4:3953. doi: 10.1038/srep03953 (PMC3912475; doi:10.1038/srep03953)
Supplement: Supplementary Information [file srep03953-s1.doc]

**Supplementary Information**

**Giant Up-Conversion Efficiency of InGaAs Quantum Dots in a Planar Microcavity**

Qinfeng Xu,^1,6^ Carlo Piermarocchi,^3^ Yuriy V. Pershin,^4^ G. J. Salamo,^5^ Min Xiao,^1,5^ Xiaoyong Wang,^1,2*^ and Chih-Kang Shih^2*^

*^1^National Laboratory of Solid State Microstructures and School of Physics, Nanjing University, Nanjing 210093, China*

*^2^Department of Physics, the University of Texas at Austin, Austin, TX 78712, USA*

*^3^Department of Physics and Astronomy, Michigan State University, East Lansing, MI 48824, USA*

*^4^Department of Physics and Astronomy and USC Nanocenter, University of South Carolina, Columbia, SC 29208, USA*

*^5^Department of Physics, University of Arkansas, Fayetteville, AR 72701, USA*

*^6^Department of Physics and Optoelectronic Engineering, Ludong University, Yantai 264025, China*

*e-mail: wxiaoyong@nju.edu.cn, shih@physics.utexas.edu

**Rate equations for the calculation of UC to DC PL intensity ratio**

For the UC process in the left panel of Fig. 5a, we can define *N*_B2_ and *N*_A1_ as the exciton populations of the B2 and A1 energy levels, respectively. Their time derivatives can be written as

$\begin{matrix} \mathop{N_{\text{B2}}}\limits^{\bullet} =P-\lambda nN_{\text{B}2}+\lambda(n+1)N_{\text{A1}}-\gamma^{'}N_{\text{B}2} \\ \mathop{N_{\text{A1}}}\limits^{\bullet} =\lambda nN_{\text{B}2}-\lambda(n+1)N_{\text{A1}}-\gamma N_{\text{A1}} \end{matrix}$ (1),

where *P* is the laser pumping rate and all the other parameters have already been defined in the text. For a steady state solution, we get

$N_{\text{A1}}(\text{UC})=\frac{P\lambda n}{\gamma\lambda n+\gamma^{'}\lambda(n+1)+\gamma\gamma^{'}}$ (2).

For the DC process in the right panel of Fig. 5a, we can similarly define *N*_B1_ and *N*_A1_ as the exciton populations of the B1 and A1 energy levels, respectively. Their time derivatives can be written as

$\begin{matrix} \mathop{N_{\text{B1}}}\limits^{\bullet} =P-\lambda(n+1)N_{\text{B}1}+\lambda nN_{\text{A1}}-\gamma^{'}N_{\text{B}1} \\ \mathop{N_{\text{A1}}}\limits^{\bullet} =\lambda(n+1)N_{\text{B}1}-\lambda nN_{\text{A1}}-\gamma N_{\text{A1}} \end{matrix}$ (3),

from which we can get a steady state solution of

$N_{\text{A1}}(\text{DC})=\frac{P\lambda(n+1)}{\gamma\lambda(n+1)+\gamma^{'}\lambda n+\gamma\gamma^{'}}$ (4).

From equations (2) and (4), we can finally obtain the UC to DC PL intensity ratio of

$\frac{N_{\text{A}1}(\text{UC})}{N_{\text{A1}}(\text{DC})}=\frac{n}{n+1}\frac{\gamma\lambda(n+1)+\gamma^{'}\lambda n+\gamma\gamma^{'}}{\gamma\lambda n+\gamma^{'}\lambda(n+1)+\gamma\gamma^{'}}$ (5).

To account for the fact that the QD coupling with B2 is about twice that with B1, we have multiplied equation (5) with a factor of 2 to get all the theoretical curves shown in Fig. 5b.


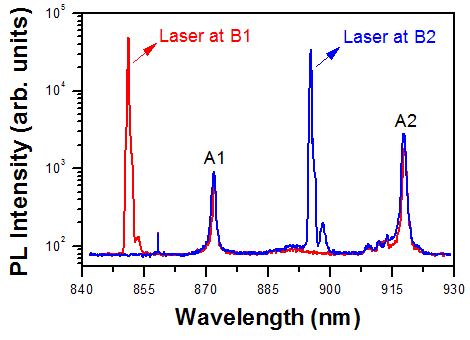


**Figure S-1.** PL spectra of the microcavity sample excited at ~120 K with the laser wavelengths at B1 and B2, respectively. When the laser wavelength was set at B1, the two DC PL peaks from A1 and A2 was observed, whose intensities roughly reflect the densities of QDs at these two energy positions. When the laser wavelength was tuned to B2 with the same power density, almost the same PL intensities from A1 and A2 were observed, as compared to those excited at B1. This confirms again that the UC PL efficiency of A1 is extremely high to be comparable to the DC PL efficiency of A2 when the excitons are created at the energy position B2 in a specific group of InGaAs QDs.


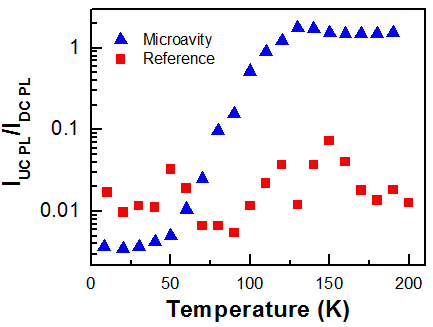


**Figure S-2.** Temperature dependences of the UC to DC PL intensity ratios for both the microcavity and reference samples plotted on a semi-logarithmic scale.


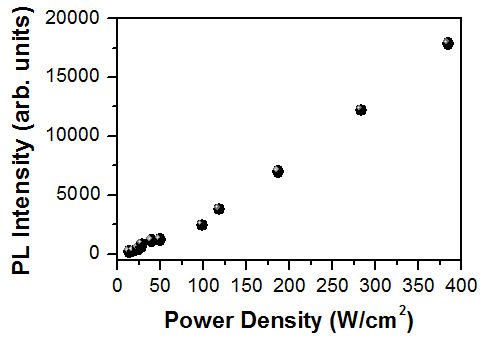


**Figure S-3.** The UC PL intensity of A1 measured at ~120 K as a function of laser power density at B2.


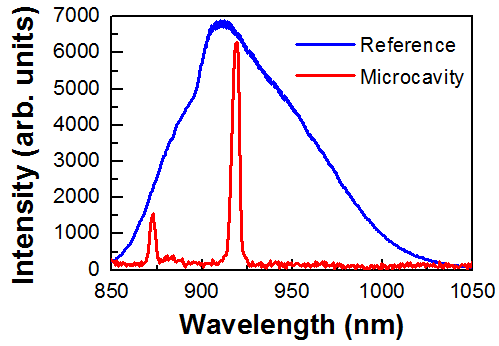


**Figure S-4.** PL spectra of the reference and microcavity samples measured at ~77 K.


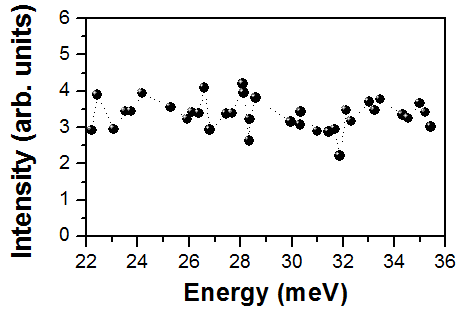


**Figure S-5.** The UC PL intensity of A1 measured at ~120 K as a function of the energy separation between A1 and B2 by changing the laser incident angles. The dotted lines are just guides to the eye.


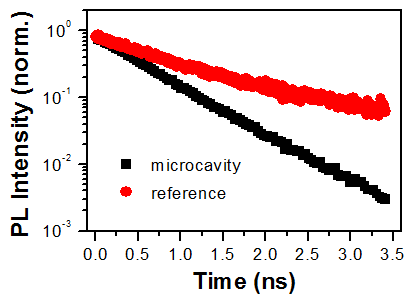


**Figure S-6.** PL decay curves measured for the microcavity and reference samples at ~8 K with the laser excitation wavelength of ~815 nm. The radiative lifetime of InGaAs QDs, which is only weakly dependent on the detection wavelength, is ~1.07 ns for the reference sample and ~0.64 ns for both A1 and A2 of the microcavity sample. This enhancement factor of <2 in the spontaneous emission rate of InGaAs QDs is expected for the vertical cavity modes of such a weak microcavity. In our theoretical calculations, we simply assume that the rate enhancement factor for A1 is equal to the rate suppression factor for B1 or B2. In reality, the rate suppression factor could be larger since it is dependent on the energy detuning from the vertical cavity modes. In the experiment, it is difficult to adjust the detection angle of the microscope objective relative to the surface normal direction of the QD sample loaded in a cryostat, so that the degree of rate suppression for the large-angle cavity modes cannot be obtained for now.
